# Radiomics in Breast Cancer: In-Depth Machine Analysis of MR Images of Metastatic Spine Lesion

**DOI:** 10.17691/stm2022.14.2.02

**Published:** 2022-03-28

**Authors:** V. Steinhauer, N.I. Sergeev

**Affiliations:** System Architect; Devoteam GmbH, 14D Wiesenstrasse, Weiterstadt, Hessen, 64331, Germany;; Leading Researcher, Department of Complex Disease Diagnosing; Russian Research Center of Roentgenology and Radiology of the Ministry of Health of the Russian Federation, 86 Profsoyuznaya St., Moscow, 117997, Russia; Professor, Department of FDPE Roentgenology and Radiology; Pirogov Russian National Research Medical University, 1 Ostrovityanova St., Moscow, 117997, Russia

**Keywords:** bone metastases, breast cancer, therapy of spine metastases, radiomics, in-depth machine analysis

## Abstract

Using mathematic criteria for image processing (radiomics) makes it possible to more accurately assess the nature of therapy-associated changes and determine the sites of maximal response. Comparison of the acquired quantitative and clinical data may assist radiologists in making the optimal decision.

**The aim of the study** was to assess the capabilities of software operators for an in-depth analysis of metastatic spine lesion images in breast cancer.

**Materials and Methods:**

MRI data of three patients with breast cancer T_2_N_2–3_M_1_ receiving treatment in accordance with the accepted clinical protocols were used in our work. Spinal metastases were assessed by a radiologist and machine analysis using the Arzela variation operators. Twelve MRI examinations (4 per each patient) excluding the baseline examination have been analyzed with a follow-up period of about 3 months.

**Results:**

The structure of the metastatically modified spine was analysed segment by segment in the sagittal and axial projections using machine image analysis operators. Rapid changes in the “complexity” of vertebrae images have been found, allowing one to suggest the efficacy of treatment in one of the three options — stabilization, improvement, progression. Changes in the vertebrae structure with a positive response to the treatment in the form of the formation of bone objects, calderas, reduction of the contrast agent circulation at the microlevel, confirmed by mathematical analysis, have been monitored. A correlation was obtained between the established changes and the level of the CA 15-3 cancer marker.

**Conclusion:**

The study has shown a high effectiveness of machine image analysis algorithms, high correlation of the obtained results with the radiologist’s report and clinical and laboratory data in 9 cases out of 12. The Pearson correlation coefficient between the classical marker and matrix filter curve was 0.8.

## Introduction

An important task of breast cancer management is the assessment of its efficacy, revealing reliable criteria of disease stabilization or progression as early as possible, which allows one to timely correct a treatment plan [1]. Application of radiomics and its principles opens wide possibilities for monitoring the disease using visualization techniques such as contrast-enhanced MRI, the advantage of which is minimal invasiveness [2, 3]. The diagnostic difficulty of these methods lies in the fact that the processes of pathologic osteolysis and reactive osteosclerosis at the microlevel are running in parallel giving a large variability of the results, especially in the process of treatment. The standard presentation of data does not answer the questions what the speed and depth of these changes are and where the maximal response to the therapy takes place. The results of radiological pattern interpretation depend directly on the radiologist’s experience and are a qualitative and semi-quantitative characteristic. The profound machine image processing using mathematical criteria will provide the possibility to obtain reliable quantitative data which may serve as a support in clinical decision-making [4]. Due to the sophisticated working process, the topic requires further exploration.

**The aim of the study** was to assess the capabilities of software operators for an in-depth analysis of metastatic spine lesion images in breast cancer.

## Materials and Methods

### Clinical description of patients

MRI data for three patients with breast cancer T_2_N_2–3_M_1_ who underwent a complex treatment including neoadjuvant chemotherapy, mastectomy, and adjuvant chemotherapy combined with external beam radiotherapy with total focal doses (TFD) up to 56 Gy. The diagnostic methods used (osteoscintigraphy, CT, and/or MRI) revealed metastatic lesions of the skeleton in all patients. Once the diagnosis has been established, MRI with intravenous gadolinium contrast enhancement was employed to further control the foci in the spine. The follow-up intervals were 3 months during which 4 examinations were performed for each woman, i.e. maximum 12 examinations. The total number of MRI investigations was 15; 3 of them represented baseline images in each observation, the rest were used to assess the effect of therapy.

### Simulation of the radiomic process

The main principle of the study has been formulated: a theoretical substantiation of the occurring processes, their practical (physical) validation, and comparison with clinical data.

Thus, the working process included two main stages: assessment of the diagnostic images by a radiologist and using machine analysis. To objectivize the results, all data were anonymized, the observations were assigned conditional numbers Osteo-1, Osteo-2, Osteo-3 with an ordinal number of examination sequence where MRI-0 is the initial examination, while MRI-1, MRI-2, etc. are diagnostic investigations made in the course of therapy. Notable, that both assessment processes were performed in parallel, and finally, a report was generated in which a final diagnosis was evaluated according to a three-valued scale: improvement, stabilization, progression. The results obtained were assessed both by simple comparison and using the Pearson correlation coefficient.

Dicom Viewer system having advanced functions of analysis and result presentation was used for machine analysis. It uses the Java programming language which makes it independent from the operating system of the computer used.

The mathematic image analysis was bound to the three main microlevel elements and included the development of the following methods:

1. analysis of the pattern complexity, since the tumor growth and angiogenesis are chaotic and therefore are characterized by a lower complexity than an ordered structure of healthy tissues;
2. contrast analysis, since contrast accumulation decreases in successful treatment;
3. analysis of microfoci (calderas), since a positive therapeutic effect in the vertebrae induces sclerotic processes at the sites of cancer lesions.

### Methods of image analysis

**Figure 1.**
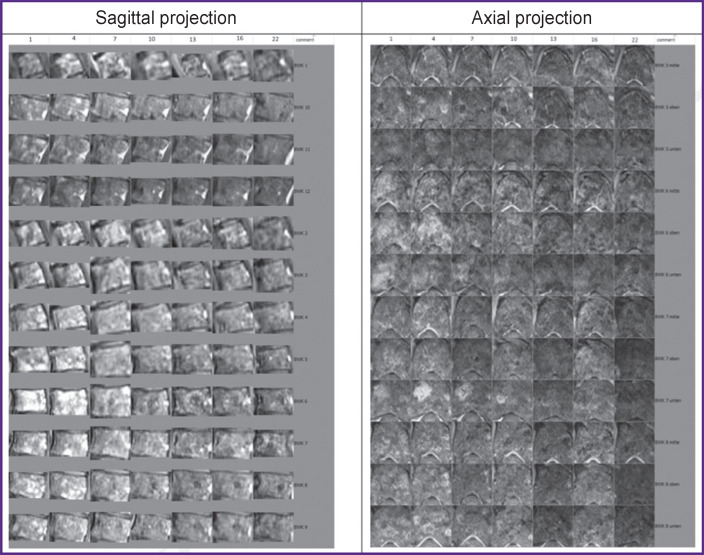
MRI in the T1-WI mode with contrast enhancement of the segmented thoracic vertebrae Th5–Th8 with a 3-month interval

Technical analysis of MR images also consisted of several stages. A series of sagittal and axial MRI in the mode of T1- and T2-weighted images (Т1-WI, Т2-WI axial, sag) with and without introduction of a contrast agent, with isolation of the vertebral body as an image segment under study. Automatization may be engaged in this process but its results are not yet sufficient despite the current technological advances [5], therefore, a manual segmentation was used in our work. Maps of change dynamics were built based on the MRI of the thoracic spine in the process of treatment (as the example, Figure 1). These maps have formed the basis for the three developed methods.

1. At the beginning of the *image complexity analysis*, some remarks should be made on the calculation of entropy. An information-theoretic approach to the assessment of image complexity as a message flow presented in a discrete form is often based on entropy computation. This variant suggests the independence of pixel values while an image of any cancer MRI pattern has a strong pixel correlation. Therefore, the approaches used to assess the complexity of independent message flow as, for example, entropy, often appear to be insufficient [6].

**Figure 2.**
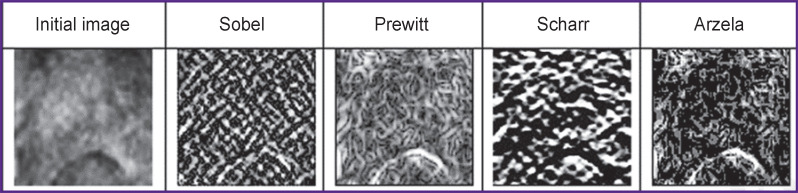
Various filters for isolation of contours on the image On images: MRI T1-WI axial with contrast enhancement, upper section of the Th6 thoracic vertebra

For this reason, in order to assess the contours or bounds (complexity) of the images, all of them were transformed to the monochrome gray color and the employed software allowed us to analyze the texture and projections simultaneously [7]. There exist several operators to calculate the object contours [8], and the most frequently used operators such as Sobel, Prewitt, Scharr, Arzela were tested by us (Figure 2).

According to the preliminary analysis, Arzela operator has displayed the best results, as it is able to show well internal contours. Arzela variations (AV) are calculated according to the following formula for each projection:

AV=∑∑|f(i+1,j)−f(i,j)|+|f(i,f+1)−f(i,j)|∑∑f(i,j),

where *f*(*i* + 1, *j*), *f*(*i, j +* 1), *f*(*i*, *j*) are gray intensity values for an indexed (*i*, *j*) pixel.

The assessment of image complexity is based on the fact that an image will be more complex than another image if the sum of the object bounds, composing it, is greater. Contrast-enhanced cancer images are less structured than the normal bone tissue due, in particular, to chaotic angiogenesis, as previously mentioned. It leads to the reduction of the contour bound length or to the smaller gradient sum, i.e. to the low image complexity. In other words, inflammation around the tumor increases the sum of contrast intensity gradients observed as angiogenesis in pathological conditions, for example, during wound healing, making a general image more complex and therefore showing the effect of therapy. Using Arzela operator we came to the conclusion that the greater AV and the higher image complexity, the closer it is to the normal tissue, be it even inflamed. AV give sufficiently good contours of the important details and may serve as a measure of complexity at least at the projections used and in the lesions seen on the spine images. The structural part (complexity) responds well to the transition from the disordered to the ordered state when the regions have average sizes from 0.5 to 2.0 cm. The susceptibility threshold is yet to be explored.

Since it is difficult to separate simultaneously a long-term contrast reception and fast changes in the axial plane at the beginning of therapy, we will enhance the effect of contrast accumulation with a weighing factor. In this way, we go to the system of weighted variations (WV): the intensity at the corresponding point *f*(*i*, *j*). WV show an integral response to the therapy, in our case the thoracic vertebrae Th5–Th8. At the initial phase, WV complexity is low but it grows sharply when active therapy is used. It is associated with the emergence of necrotic areas where the structure of the contrast agent accumulation is more complex than in the tumor region. The process transforms then to the stable phase, the complexity of which (in the direction of the normal tissue) is substantially higher than in the initial phase.

**Figure 3.**
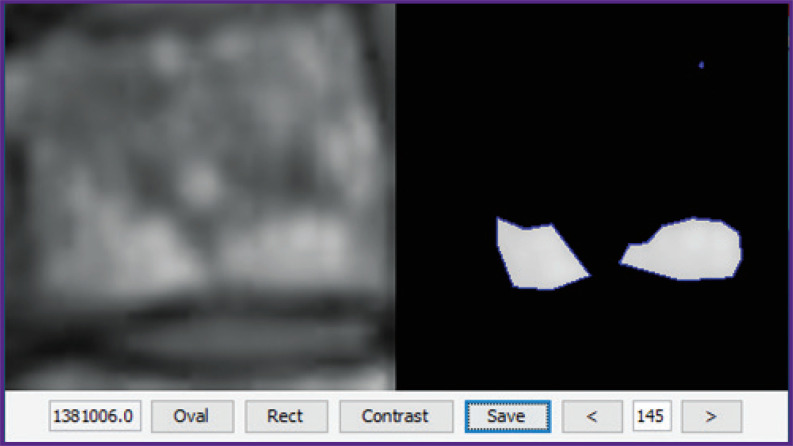
Automatically isolated contours (BoofCV) for Th7 thoracic vertebra on T1-WI sag with contrast enhancement

2. *Automatization of contrast measurement*. Automatic detection of high contrast areas (bright areas) was used. After several experiments, the following simple algorithm was obtained: extrapolation of segmentation objects (usually about 100×100 pixels) with a cubic spline up to the size of 250 pixels; calculation of the threshold values for the average image brightness, and finally, contouring using BinaryImageOps.contour function from the BoofCV library with subsequent calculation of the total intensity in the contours (Figure 3).

The area of contours (in pixels) is summed up in order to take into consideration the influence of intensity distribution within the contours.

Contact=∑∑P(i,j),

where *P*(*i*, *j*) is the pixel value (0–255); *i* is the contour index; *j* is the number of pixels in the contour.

As a result, a quantitative curve of the contrast agent reception has been obtained as a function of a prolonged time over the entire registered spine region.

3. *Microfoci*. When moving to the last stage, we proceeded from the previously performed pathological studies [9], which have established that objects with radial and spherical symmetry prevailed in breast cancer. Keeping this in mind, we were searching for the lesion objects with this symmetry on the MR images of the thoracic spine. At this stage, we faced the questions of differential diagnosis which require a large separate investigation. In our work, enostosis-like changes have been detected: they had the same annular shape but with greater homogeneity [10].

At the first stage of our work, a standard conclusion was formulated by a radiologist with the assessment of the ongoing visible processes. Moreover, the protocol was more detailed owing to the possibility to trace the changes over the long follow-up period using available means of image processing on the workstations such as magnification and comparison.

**Figure 4.**
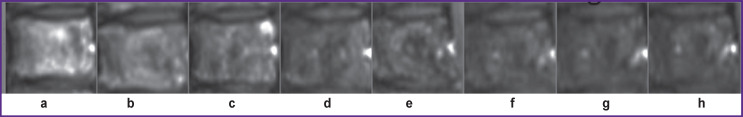
Caldera development from angiogenesis to sclerosing; MRI T1-WI sag with contrast enhancement

Figure 4 shows visible changes on MR images (contrast-enhanced T1-WI sag) occurring with time for thoracic vertebra Th6:

- developed tumor: randomly located blood vessels superiorly and inferiorly with intensive accumulation of the contrast agent;
- destruction of the tumor angiogenesis and inflammation: formation of caldera;
- randomly located vessels are almost absent in the lesion, caldera is growing and structuring;
- an “inflammatory rim” appears around caldera, its structuring continues;
- the inflammatory rim is replaced with a “sclerotic” ring, the caldera’s bottom becomes more homogeneous, one more reception of the contrast agent takes place;
- the caldera closes in the center, the rim is well-seen;
- the caldera loses its shape and merges further with the general background;
- further stabilization of the process occurs.

The same conditions are observed in other vertebrae. Several similar processes may run on one vertebral body and not always simultaneously.

**Figure 5.**
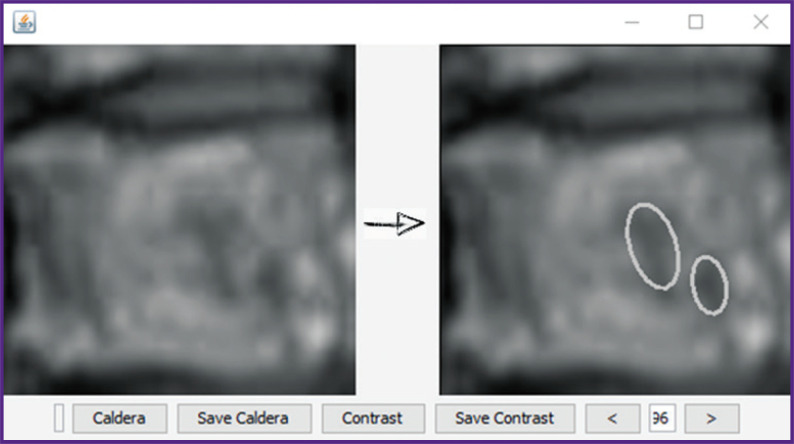
Example of detecting elliptic calderas using BoofCV software package

In the course of the work, it was suggested to call “calderas” all obscure spherically and radially symmetrical objects (microfoci). Before identification of calderas, images were stretched up to the size of the internal standard (250×250) and then smoothing was performed. For detection, it was necessary to set a threshold which varied from 1 to 254. Then a mean weighted value was counted which was taken as a threshold where the weights are the values of caldera areas. About 20% of the image bounds which were beyond the vertebra surface were excluded from the calculations. An example of calderas identification by the system is presented in Figure 5.

A quantitative value for the caldera was counted as a total caldera area on the examined part of the spine:

Caldera=∑S(i),

where *S*(*i*) is the area of separate calderas. This value is expressed in percentage in relation to the examined part of the spine surface.

## Results

On the first point, complexity assessment, calculations were done concurrently for separate vertebrae and for the group of vertebral bodies.

**Figure 6.**
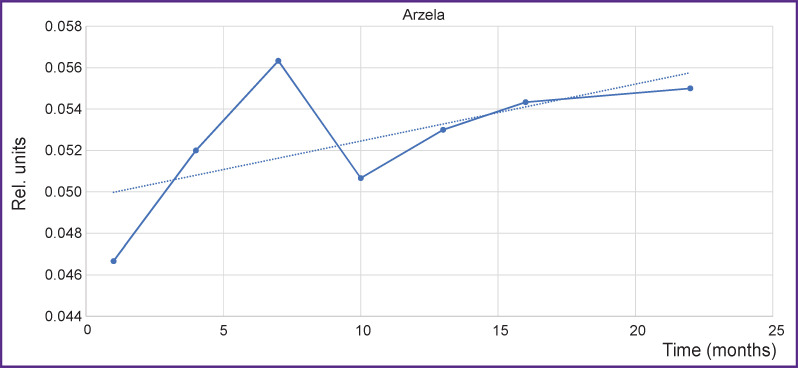
Changes of Arzela variations for Th7 thoracic vertebra on T1-WI axial with contrast enhancement *Solid line —* Arzela variations; *dotted line* — tendency to Arzela variation increase. Horizontal axis corresponds to the time from the start of observation

Having calculated the values of variations, let us consider the changes of AV on the axial projection using the seventh thoracic vertebra Th7 as an example (Figure 6).

It is seen how AV (complexity) grows not only on the initial region but asymptotically as a whole, moreover, some spread in values is quite possible due to the technical parameters of MRI. The first values show the most sensitive response to the therapy and may serve as an additional landmark for the correction of the treatment scheme.

**Figure 7.**
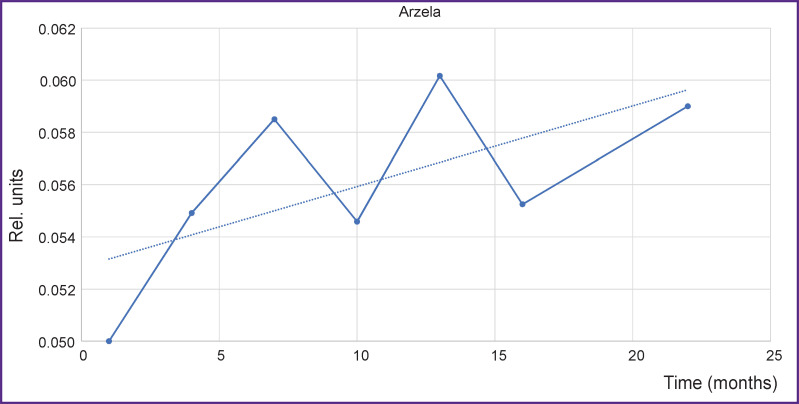
Changes of Arzela variations over time simultaneously for thoracic vertebrae Th5– Th8 on T1-WI axial with contrast enhancement *Solid line —* Arzela variations; *dotted line —* tendency to Arzela variation increase. Horizontal axis corresponds to the time from the start of observation

To improve statistical results, various segments of several vertebrae have been analyzed concurrently, smoothing thereby statistical dispersion. The results of AV for a large volume including Th5–Th8 vertebrae are presented in Figure 7.

The curve behavior for a large volume of vertebrae is seen to be similar to that for separate vertebrae, which leads to the important conclusion: the increase of the reference values may be used for a rapid assessment of the therapy. Besides, asymptotic behavior (*dotted line*) may be employed for revealing the cumulative therapy effect in a long-term perspective.

The result of AV calculation on the sagittal projection without a contrast agent (T1-WI sag and T2-WI sag) appeared to be somewhat unexpected. At the beginning of the study, the axial projection of the vertebral surfaces was supposed to be the most sensible, which was actually confirmed. However, the selectivity of the tumor was lower than in the sagittal projection. After verification of this theory, the results will be presented in our subsequent works.

**Figure 8.**
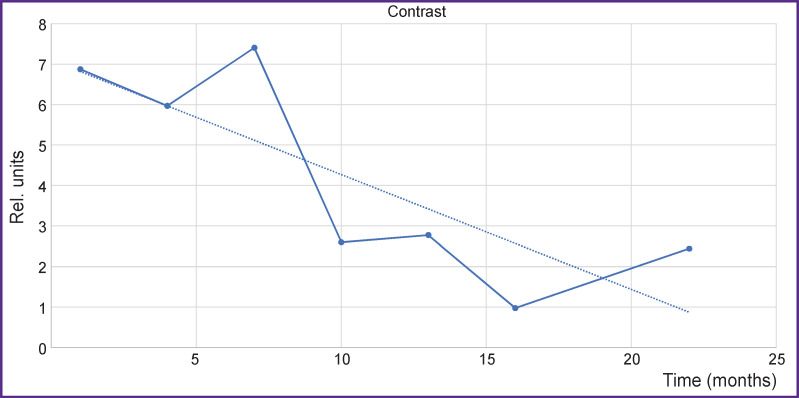
Change in the contrast agent accumulation over time (horizontal time axis) on T1-WI sag with contrast enhancement *Solid line* — filling with contrast, decrease is noted; *dotted line —* tendency to the decrease of contrast agent accumulation

When measuring the contrast on T1-WI sag with contrast enhancement, we got a quantitative curve of the contrast agent reception as a function of prolonged time over the entire registered region of the spine (Figure 8).

**Figure 9.**
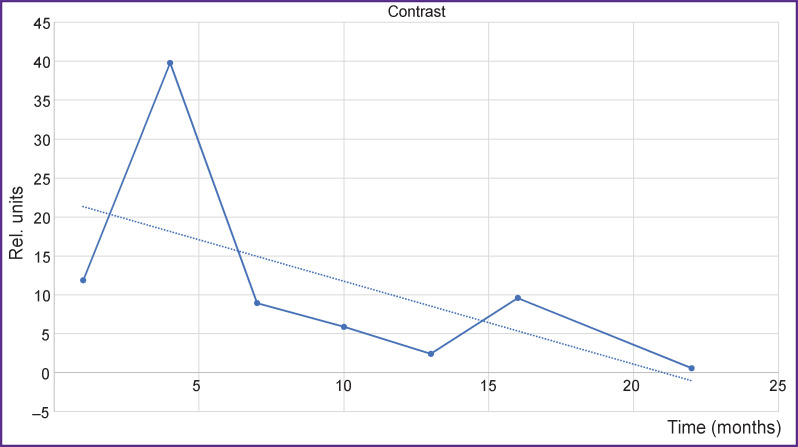
Change in the contrast agent accumulation over time shown by the data of T1-WI axial with contrast enhancement *Solid line —* filling with contrast, decrease is noted; *dotted line —* tendency to the decrease of contrast agent accumulation

The data on the contrast accumulation were checked in the axial projection using the same method and unchanged settings: the threshold values of the mean image intensity (gray value) (Figure 9).

Comparison of the curves in the axial and sagittal projections shows that they do not essentially change their local behavior although they preserve all global properties. Thus, we observe a drop in contrast accumulation up to some asymptotic value in our case. This analysis of contrast accumulation allows us to quantitatively evaluate long-term changes of the process activity in the tested zone (vertebral bodies) according to MRI data.

### Microfoci

**Figure 10.**
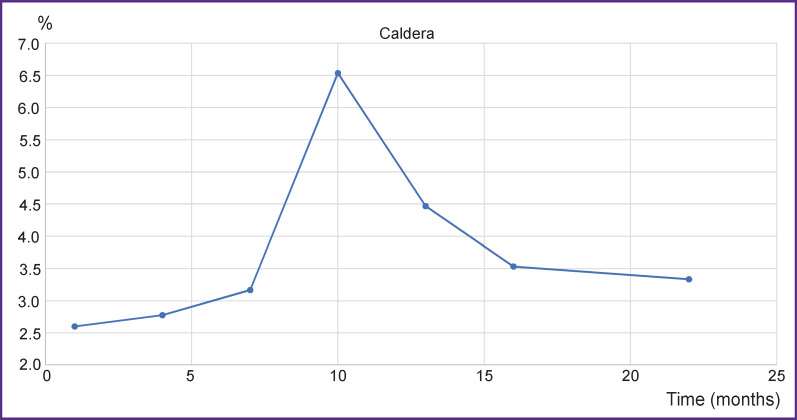
Caldera changes over time according to the data of T1-WI sag with contrast enhancement Caldera values are expressed in percentage to the area of the vertebrae projection

The final result of microfoci analysis, as mentioned above, is summing up all caldera values for all thoracic vertebrae. Figure 10 shows a diagram where maximum corresponds to spherically or radially symmetrical rim-forming objects and, what is most likely, with suppressed foci. Thus, about 10 months after the beginning of therapy, maximal suppression of the foci is achieved and over time this process is replaced with sclerosing of the niches formed.

### Data interpretation

A high degree of coincidence between the estimates made by the radiologist and machine analysis system has been achieved: 9 of 12. In two cases, the radiologist’s conclusion “stabilization” disagreed with the system’s assessment “progression”, subsequent follow-up showed worsening of the condition. In one case, the radiologist’s “stabilization” differed from the system’s “improvement” due to the higher complexity of the objects. Osteo-1 follow-up report is presented as an example (see the Table).

**Table:** Conclusions made by the radiologist and machine analysis system for Osteo-1 observation

Examinations	Radiologist’s conclusion	System’s conclusion
MRI-0 (initial)	Multiple metastatic lesion of the thoracic spine. Pathologic fractures are not revealed	Low complexity
MRI-1	Reduction of trabecular edema zones, decrease of paramagnetic accumulation degree	Complexity growth: positive response to therapy
	***Improvement***	***Improvement***
MRI-2	Reduction of trabecular edema zones, formation of “dark” regions of osteosclerosis	Intensive complexity growth according to axial and sagittal data: positive response to therapy
	***Improvement***	***Improvement***
MRI-3	MRI image without essential dynamics, absence of new foci: process stabilization	Sharp reduction of complexity in both projections: a sign of progression
	***Stabilization***	***Progression***
MRI-4	Emergence of new metastatic foci, enlargement of previously detected foci in size	Continuing reduction of object complexity in both projections
	***Progression***	***Progression***

**Figure 11.**
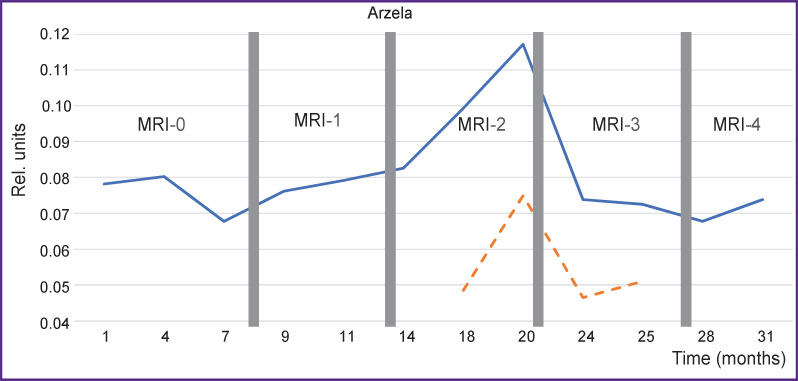
Complexity changes for the Osteo-1 patient over time *Solid line —* according to the Т2-WI axial data; *dotted line —* Т2-WI sag, complexity increase is noted in the average observation interval with its subsequent sharp reduction

To build the dependence graph for changes, 50 axial slices of MRI T2-WI were used, about 600 measurements altogether (Figure 11).

**Figure 12.**
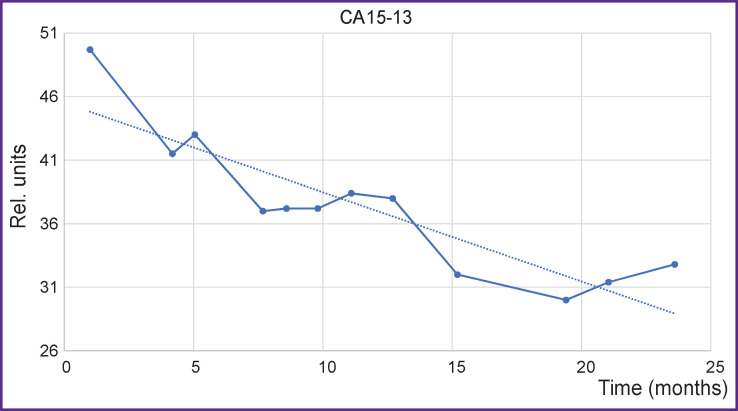
Change of the tumor marker СА 15-3 from the 1^st^ to 22^nd^ month of MRI follow-up *Solid line —* CA 15-3; *dotted line —* tendency to the marker changes

To illustrate the possibilities of the additional control of the therapy process, changes in the classical indicator, tumor marker CA 15-3, are presented for the case of the established tumor regression (Figure 12). Values of the marker changes were assessed in compliance with the accepted clinical recommendations [11]. CA 15-3 is seen to correlate well with AV, Contrast, and Caldera in a long-term perspective. The Pearson correlation coefficient between AV and CA 15-3 was 0.8 in this case.

## Discussion

Summing up the work, we would like once again to point to a very important fact for us and other researchers, which was the basis of our line of reasoning: at the microlevel, metastatic lesion is usually presented by a round or oval defect in osteoclastic variant of breast cancer, the effect of radial or spherical symmetry altering under the influence of therapy is also observed [12]. In the course of treatment, the graphic image of the metastatic lesion is changing. The most important diagnostic sign of a positive trend in treatment is the increase of the bone structure density at the lesion site [13]. A wide coverage of our algorithm (it works in different kinds of bone metastases) is explained by the fact that the first manifestation of reparation in osteoclastic metastases, i.e. appearance of a sclerotic rim around the lytic focus and subsequent gradual filling of the focus with the bone tissue, occurs in 93% of patients during 3–6 months after the beginning of the successful treatment [14].

With the help of the operators used in our study, we managed to trace the response to the therapy of breast cancer with bone metastases on the three similar clinical cases. We understand that it is not sufficient to make unambiguous conclusions; therefore, we tried to upgrade the reliability analyzing simultaneously several vertebrae in several MRI modes. We are going to extend the presented algorithm to other MRI sequences and to the CT data as well. In the process of our work, we also managed to define several limitations of the methods used.

## Conclusion

The investigation performed has shown a high percentage of agreement between the results obtained by radiologists and by means of machine analysis when analyzing the state of breast cancer with metastatic spine lesion (Pearson correlation coefficient reached 0.8 for the case of regression). This approach may be used as a support in clinical decision-making although the this process itself requires further development, first of all, in the direction of classification of the results with the help of neural networks as we have used it previously for automatic leukocyte classification [15].

The machine analysis system allows one to achieve a deeper understanding of the interaction between morphology and process characteristics in metastatic breast cancer, to assess more fully the effect of therapy.
